# Trial-based economic evaluation of the system-integrated activation of community health volunteers in rural Ghana

**DOI:** 10.1080/16549716.2023.2203541

**Published:** 2023-05-11

**Authors:** Yinseo Cho, Koku Awoonor-Williams, Damin Jun, Chunghyeon Oh, Seungman Cha

**Affiliations:** a West Africa Division, Korea International Cooperation Agency, Seongnam-si, Korea; b Policy, Planning, Monitoring and Evaluation Division, Ghana Health Service, Accra, Ghana; c Performance Management Team, Korea International Cooperation Agency, Seongnam-si, Korea; d Fiji Office, Korea International Cooperation Agency, Suva, Fiji; e Department of Surgery, CWM Hospital, Suva, Fiji; f Department of Global Development and Entrepreneurship, Graduate School of Global Development and Entrepreneurship, Handong Global University, Pohang, South Korea; g Faculty of Infectious and Tropical Disease, London School of Hygiene & Tropical Medicine, London, UK

**Keywords:** Community health volunteers, Ghana, economic feasibility, cost benefit, cost effectiveness

## Abstract

**Background:**

Globally, steps to revitalise programmes deploying community health workers (CHWs) on a national scale have been growing, but few economic evaluations have been done on system-integrated CHW programmes. Ghana has dual cadres of CHWs: community health officers (CHOs) and community health volunteers (CHVs). CHO plays a major role in primary health services but has suffered from chronic staff shortages. We activated CHVs in communities to mitigate the negative impact due to CHO shortages. The CHVs conducted home visits and provided health education to prevent childhood diseases.

**Objective:**

We evaluated the cost-effectiveness and cost-benefit of activating CHVs.

**Methods:**

In a cluster-randomised trial with 40 communities in rural Ghana, the changes in disease incidence were inferred from a statistical model using a Bayesian generalised linear multilevel model. We evaluated the total incremental cost, benefit, and effectiveness for the intervention from an economic model. In cost-effectiveness analysis, disability-adjusted life years (DALYs) were estimated using a decision tree model. In the cost-benefit analysis, the cost-benefit ratio and net present value of benefit were estimated using a decision tree model, and a standardised sensitivity analysis was conducted. The decision tree model was a one-year cycle and run over 10-years. Costs, benefits, and effectiveness were discounted at a rate of 3% per year.

**Results:**

According to the cost-effectiveness analysis, the programme was highly likely to exceed the WHO-CHOICE threshold (1–3 times GDP per capita), but it was unlikely to exceed the conservative threshold (10–50% of GDP per capita). In the cost-benefit analysis, the mean and median cost-benefit ratios were 6.4 and 4.8, respectively.

**Conclusion:**

We found the potential economic strengths in the cost-benefit analysis. To integrate CHW programmes with national health systems, we need more research to find the most effective scope of work for CHWs.

## Background

The recent global consensus that community-based primary healthcareCBPHC) is the single most important means of achieving universal health coverage (UHC) and the Sustainable Development Goals has been re-visited [1]. In 2018, the global health community approved the Astana Declaration, reaffirming that UHC cannot be achieved without strong support from CBPHC [2]. In 2019, the UN General Assembly adopted a historic resolution on the commitment of all the member states to achieve UHC by 2030 [3].

However, the resolution also warned that one-third of the world’s population will not have access to essential healthcare 2030 if the current pace of progress remains unchanged [3]. One of the reasons that the pace has stagnated is due to the shortage of essential healthcare workers. Globally, 18 million additional health workers are needed to ensure effective coverage of essential health services. This problem is most serious in Africa, which is the only continent that will suffer from a health worker shortage by 2030 due to its rapid population growth. According to the World Health Organization (WHO), Africa’s health worker shortage is estimated to increase by 36%, from 4.5 million in 2013 to 6.1 million in 2030 [4].

Correspondingly, interest in revitalising national programmes involving community health workers (CHWs) is growing. Several case studies have demonstrated that community-based interventions, ranging from family planning to neonatal survival, can be effectively delivered by CHWs in low- and middle-income countries [5]. Moreover, after the recent public health crisis of COVID-19, the argument has been made that CHWs are also needed in health systems in high-income countries [6].

Nonetheless, many existing CHW programmes are small scale, run by nongovernmental organisations, and not integrated into national health systems, and in many cases, these programmes fail to be scaled up [7]. To expand a CHW programme to a national scale, it is necessary to conduct an economic evaluation of the relative value of investments in the presence of an improved CHW programme within the health system to obtain more robust evidence [8]. To date, there have been few economic evaluations of CHW programmes integrated into national health systems [9].

Ghana has a long history of integrating CHWs into its primary health system, called Community-Based Health Planning and Services (CHPS), making it a suitable country for evaluating system-integrated CHW programmes [10]. Ghana’s CHPS system is run by the dual cadres of CHWs: community health officers (CHOs) and community health volunteers (CHVs) [11]. CHOs are government-paid full-time nurses residing near the community, while CHVs are part-time volunteers residing in the community. CHOs receive 2 years of training in nursing school, while CHVs receive 1–2 weeks of workshop-based training. In principle, CHOs serve 3,000–5,000 residents in 5–8 communities (named a CHPS zone), and CHVs support CHOs. Both CHOs and CHVs are recruited, trained, rewarded, and monitored by the regional team of the Ghana Health Service under the Ghanaian government. In 2016, Ghana had 2,523 CHOs and 19,411 CHVs across 5,062 CHPS zones [12].

Regular home visits are among CHWs’ primary CBPHC interventions. Home visits are related to health promotion and surveillance across various topics, ranging from newborns to non-communicable diseases [7]. In Ghana, home visits have been emphasised since the introduction of the CHPS system [10]. Ghana’s health policy requires CHOs to visit all households in the catchment area every quarter.^11^ However, adherence to home visits is poor. According to some surveys, 27–70% of the respondents said they had never had a home visit in the past 3 months [13,14].

We implemented a method of activating CHVs to conduct home visits in Volta, a region rarely visited by CHOs, and evaluated whether it contributed to childhood disease prevention. Our study aimed to evaluate the causal effect of activating CHVs for childhood disease prevention and its economic feasibility from a societal perspective through a field experiment. We performed cost-effectiveness and cost-benefit analyses of CHV activation in Ghana’s primary healthcare system. The health impact evaluation of our field experiment has been previously published, and this economic evaluation is a follow-up study to the previous health impact evaluation.

## Methods

### Trial design

From 1 February 2015, to 20 September 2016, a cluster randomised trial was conducted to measure the causal effect of the intervention on the reduction of diarrhoea and high fever among children (ISRCTN49236178). Our study area covered 40 communities in the Ketu South district in the Volta region of Ghana. The study population was 24,765 people from 4,953 households. Systematic sampling was applied to select study participants in the clusters (communities). The data collectors visited households in each community using the interval method, in which the total number of households in a community is divided by the cluster size. They started by visiting a household located nearest to the main road, from which they continued to visit the next nth household based on the interval (e.g. the next fifth household if the interval was 5). They asked any household members if they had at least one under-5 child. If the household did not meet the eligibility criterion, the household was excluded, and the data collectors continued visiting the next nth one.

The primary outcomes of the trial were the period prevalence (proxy measurement of cumulative incidence) of diarrhoea and high fever (proxy measurement of malaria) among children under 5 years of age. Data were collected by conducting a longitudinal panel survey three times (at 0, 6 and 12 months). We adopted a phase-in design in which CHVs were first activated for 20 communities in the intervention group, and CHVs were activated for 20 communities in the control group after the end-line survey was completed. Details of the trial design are provided in the study protocol of the supplementary material #2.

### Intervention

The intervention comprised the activation of CHVs. CHVs’ core task was to visit each household once every 2 months. They were recommended to spend at least 30 minutes on each visit. During the visits, CHVs were instructed to use visuals to provide health education on 10 key messages. The messages included information on preventing diarrhoea and malaria: specifically, stating that proper hand-washing and improved hygiene and sanitation can prevent diarrhoea and sleeping under an insecticide-treated mosquito net can prevent malaria. CHVs were also encouraged to support CHOs and mobilise community members to attend child welfare clinics, which were held on a monthly basis in the community.

We worked to integrate our interventions into the national health system and minimise external interference. CHVs were recruited by community committees based on their literacy, volunteerism, and work experience. Ghana Health Service’s District Health Management Team coordinated the recruitment process according to national guidelines. The CHVs were trained for 5 days before being deployed. During the training, CHVs learned the basics of essential child healthcare as well as the concepts, roles, and responsibilities of the CHPS system, and reporting and recording skills.

## The framework of economic evaluation

**Figure 1.**
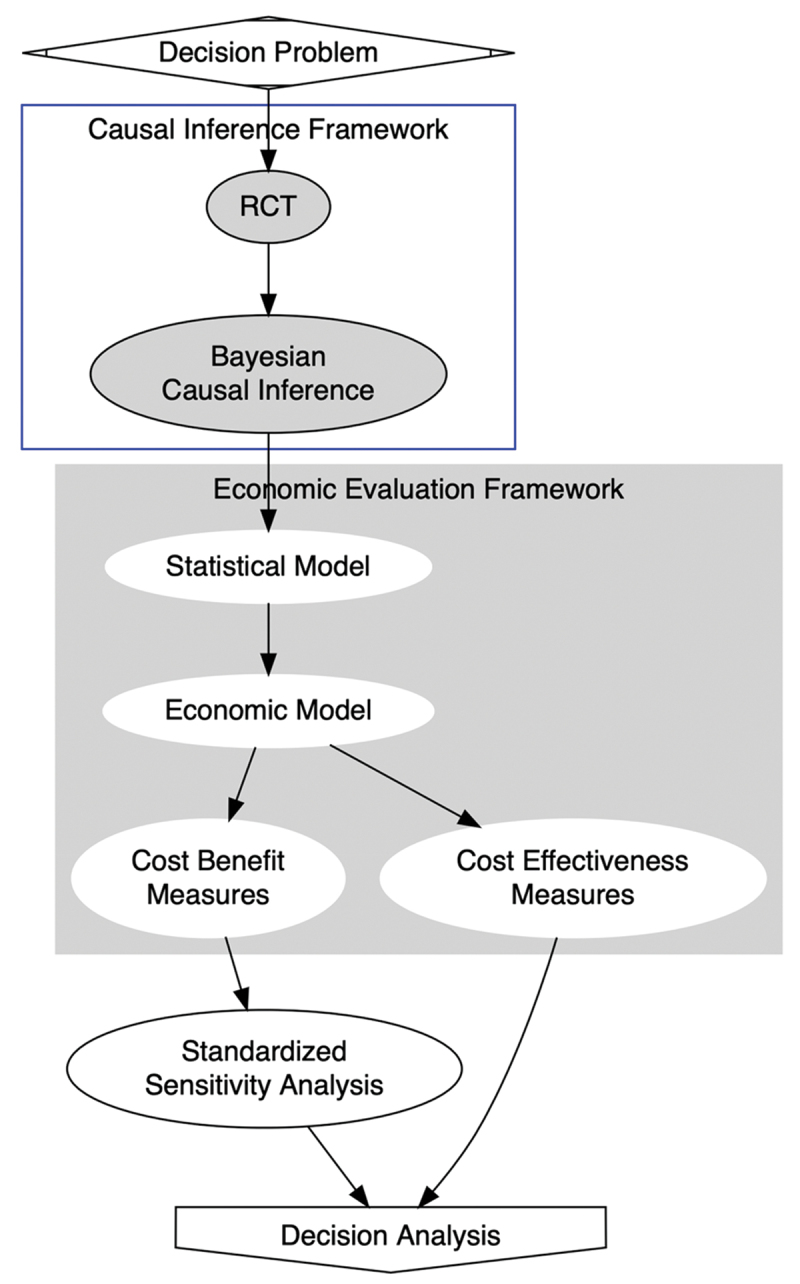
The framework of the trial-based economic evaluation.

Figure 1 presents a graphical representation of the framework that we used. We combined the frameworks of causal inference and economic evaluation for estimating the economic consequences of the intervention rigorously. For the causal inference framework, we applied a Bayesian approach estimates the average treatment effect of the intervention with the BRMS package in R [15]. For the economic evaluation framework, we adopted Bayesian methods for health technology assessment proposed by Gabrio et al. [16] As depicted in Figure 1, we formulated the decision problem first. We then inferred the causal effects of the intervention on reducing diarrhoea and malaria, which are two major childhood infectious diseases in Ghana. The causal effects were inferred as probabilistic samples that represented the intervention outcomes, and these samples were then used as inputs for the economic models that evaluated the Cost-Effectiveness and the Cost-Benefit metrics. The Cost-Effectiveness metrics comprised Incremental Cost-Effectiveness Ratio (ICER) and Cost-Effectiveness Acceptability Curve (CEAC). The Cost-Benefit metrics consisted of Cost-Benefit Ratio (CBR) and Net Present Value for Benefit (NPVB). From the Cost-Effectiveness Analysis, we estimated the density of ICERs by plotting the Cost-Effectiveness Plane. The dots on the plane represented probabilistic samples of incremental effectiveness (x-axis) and incremental cost (y-axis). A shaded part of the plane indicated the area that 1 DALY was averted with a cost less than Ghana’s GNI per capita. We plotted CEAC, in which we illustrated five different cost-effectiveness thresholds (CETs) reflecting a guidance of WHO-CHOICE study and a recent study on optimal cost-effectiveness thresholds for low- and middle-income countries [17,18]. For the Cost-Benefit Analysis, we draw violin plots to explain the results of the standardised sensitivity analysis. The advantage of violin plots is that they show the entire distribution of estimates and can deal with a multimodal distribution with more than one peak. Table 1 shows the six scenarios of the standardised sensitivity analysis reflecting a suggestion by Robinson et al. [19] Finally, we conducted decision analysis to support the policymaking process for community health worker programmes in Ghana and other low- and middle-income countries.

**Table 1.** The scenarios of standardised sensitivity analysis (SSA).

	Unadjusted Life Expectancy (Ghana’s average age)	Adjusted Life Expectancy (2 years-old)
VSL = Ghana’s GNI per capita × 160	SSA 1	SSA 2
VSL = Ghana’s GNI per capita × 100	SSA 3	SSA 4
VSL = (Ghana’s GNI/US’s GNI)^1.5(income elasticity)^× US’s VSL	SSA 5	SSA 6

* VSL = Value of Statistical Life; GNI = Gross National Income.

### Statistical model

**Figure 2.**
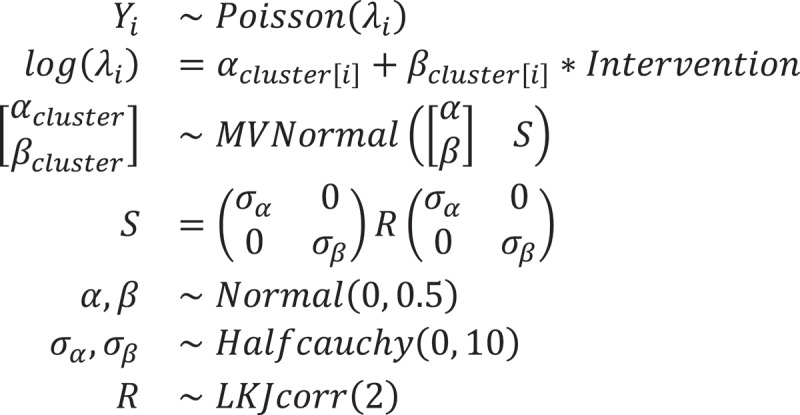
Statistical model for Bayesian inference.

This economic evaluation used Bayesian inference to estimate the causal effects of the trial, but the previous health impact evaluation of the same trial used a frequentist’s approach.^15^ In Figure 2, our target parameter was the rate ratio of childhood diarrhoea and high fever period prevalence (proxy measurement of relative risk) between the intervention and control groups. Bayesian inference is a process of inferring the probability distribution of parameters of a target population from data using Bayes’ theorem [20]. We used the credible interval for estimating the trial effects, which is comparable to the confidence interval in frequentist statistics. We interpreted the credible interval in which the true estimate (relative risk between the groups) would lie within the interval in 95% probability, given the observed RCT data.

We used a Poisson generalised linear multilevel model (GLMM) to analyse the data. The Poisson model is an appropriate method to infer relative risk by comparing the incidence of two groups based on count data. We also adopted a multilevel model that used random mixed effects in clusters to align the trial characteristics and the statistical model [10,21]. We also used weak-informative prior distributions for the target parameters.

### Economic model

**Figure 3.**
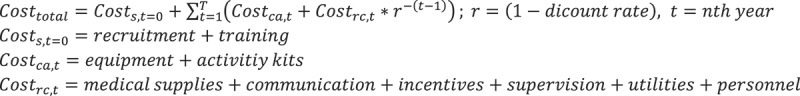
Economic model for cost.

We deduced the total incremental cost of activating CHVs in the health system over the next 10-year period. In Figure 3, we calculated three types of direct financial costs. The start-up cost ($Cost_{s,t=0}$) is a one-time investment at the beginning stage, for purposes such as recruiting and initial training. Capital cost ($Cost_{ca,t}$) refers to elements that have remaining values over time, such as vehicles and equipment. The recurrent cost ($Cost_{rc,t}$) includes expenses that reoccur every year such as consumables. To measure financial costs precisely, we retrospectively extracted micro-cost data from the expenditure records of the project. We verified the quality of the cost data by consulting its validity with project staff.

In addition to financial costs, we also reflected various economic costs in our model, including the time spent by caregivers, CHVs, CHOs, the monitoring staff of Ghana Health Service, and project consultants. Finally, the total incremental cost was converted to a constant 2016 US dollars ($1 = GHS 3.91) and discounted annually by 3% [17]. We modelled the probabilistic uncertainty of the total cost by using a log-normal probability distribution, assuming the standard deviation as 25% of the mean value. Table 2 briefly shows the values of the cost parameters that we used in the economic model for cost calculation. More specific information on the cost parameters was attached to the supplementary material #1.

**Table 2.** Parameters for economic model (cost).

Type	Account	Life span (Year)	Unit Cost (US$)	Unit Cost (GHS)	Total Cost (US$)
Initial cost	CHV recruitment	Once	8,670	33,900	10,102
Initial cost	CHV initial training	Once	1,024	4,005	10,524
Capital cost	Equipment	5	40,000	156,400	40,000
Capital cost	CHV activity kits	2	132	518	10,879
Recurrent cost	CHV consumables	1	10	41	860
Recurrent cost	Medical supplies	1	184	720	15,099
Recurrent cost	Communication	1	46	180	1,872
Recurrent cost	Motivation	1	1,545	6,044	14,925
Recurrent cost	Supervision	1	2,635	10,305	18,841
Recurrent cost	Administration	1	1,491	5,833	17,902
Recurrent cost	Personnel	1	50,837	198,773	100,280

* More detail information on the parameters is included in the supplementary material #1.

**Figure 4.**
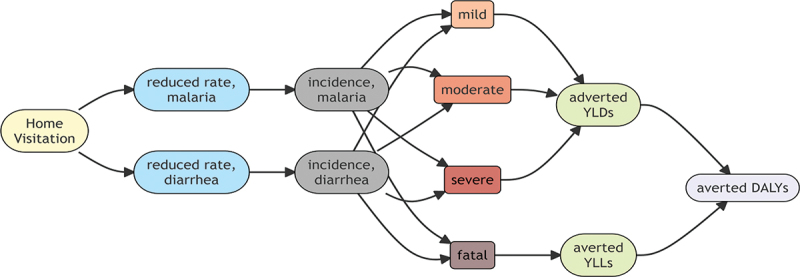
Economic model for effectiveness.

In Figure 4, the economic model for the expected incremental effectiveness, we created a decision tree model for the one-year cycle and ran the model over 10-years by computing probabilities through expected causal paths from reduced relative risk inferred from the statistical model to the amount of disability-adjusted life years (DALYs).

First, in the statistical model, the relative risk of diseases between the two groups was inferred, and the relative risk reduction was derived by subtracting 1 from the relative risk. Second, the reduced disease cases in the target population (intervention group) were calculated from the combined probability of the relative risk reduction and the average incidence of diseases in the intervention group. Third, we calculated the number of averted DALYs in the target population during a 1-year cycle by proportionally shifting the reduced disease cases to mild, moderate, severe, and fatal states. Finally, the total expected incremental DALYs over the 10-year period was calculated by applying the annual population growth rate of 2.2%, the discount rate of 3% and the CHVs’ attrition rate of 8.3% to the 1-year cycle economic model for ten times. We estimated the attrition rate of CHVs based on field research in rural Ghana finding that after activating CHVs, they began to deactivate at an attrition rate of 8.3% per year and did not remain active in the community after 10 years.^21^ The rates of population growth and discount were obtained by referring to the recent World Bank dataset and WHO guideline [17,22].

We evaluated the incremental cost-effectiveness of our model with the incremental cost-effective ratio (ICER) per DALY and the probability of accepting the cost-effective thresholds (CETs). We set up five CETs to reflect variation in policymakers’ willingness-to-pay. The first and second CETs (20% and 50% of GDP per capita) were selected with reference to previous studies investigating CETs based on opportunity costs in low- and middle-income countries [18]. The third to fifth CETs (1 to 3 times GDP per capita) were selected by referring to the widely used WHO-CHOICE study [17]. Tables 3 and 4 briefly show the values of the parameters that we used in the economic model for the effectiveness and benefit calculation. The detailed information on the parameters was attached to the supplementary material #1.

**Table 3.** Parameters for economic model (probabilistic).

Name	Mean	Upper	Lower	Type
Disability weight for diarrhea with mild severity	0.074	0.104	0.049	beta
Disability weight for diarrhea with moderate severity	0.188	0.264	0.125	beta
Disability weight for diarrhea with severe severity	0.247	0.348	0.164	beta
Disability weight for malaria with mild severity	0.006	0.012	0.002	beta
Disability weight for malaria with moderate severity	0.051	0.074	0.032	beta
Disability weight for malaria with severe severity	0.133	0.190	0.088	beta
Diarrhea Incidence	2.055	2.443	1.675	lognormal
Malaria Incidence	0.433	0.582	0.264	lognormal
Life expectancy at 2 ages	65.9	67.2	64.5	Normal

**Table 4.** Parameters for economic model (constant).

Name	Value	Name	Value	Name	Value	Name	Value
Transition probability of diarrhea (mild)	0.624	Transition probability of diarrhea (moderate)	0.289	Transition probability of diarrhea (severe)	0.042	Case Fatality Rate of diarrhea	0.0005
Transition probability of malaria (mild)	0.500	Transition probability of malaria (moderate)	0.300	Transition probability of malaria (severe)	0.200	Case Fatality Rate of malaria	0.024
Duration of diarrhea (days)	4.2	Duration of malaria (days)	7	CHV’s annual attrition rate	0.083	Discount Rate	0.03

**Figure 5.**
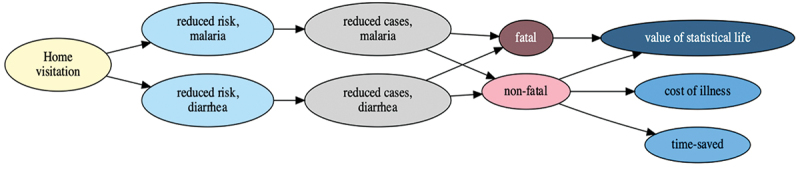
Economic model for benefit.

In Figure 5, the economic model for the expected incremental benefit, both health and non-health outcomes, were evaluated as the monetary value of the expected incremental benefits. For health benefits, we calculated the number of fatal and non-fatal disease cases prevented by home visits. The fatal and non-fatal cases were evaluated by the statistical life value (VSL) and cost of illness. For non-health benefits, CHOs’ time value was calculated by multiplying the saved time by the average hourly wage [23]. Since Bayesian inference directly infers the underlying probabilistic samples of the target parameters, the probabilistic sensitivities of the parameters are fully reflected without sensitivity analysis. However, an additional sensitivity analysis was conducted to deal with uncertainties in the benefit model, such as income elasticity and indirect VSL estimation. We conducted a standard sensitivity analysis of the benefit model with reference to the recent guide to cost-benefit analysis for global health [24]. We adopted societal perspective for all analyses, and all reproducible codes for this study can be found at https://osf.io/xsvwr/.

## Results

Using a Bayesian GLMM statistical model, we analysed how childhood disease incidence changed through the baseline, mid-line, and end-line longitudinal panel data. We found that compared to the control group, the incidence of the outcomes of interest among children under 5 in the intervention group changed to a 95% credible interval of 0.661–1.128 (median: 0.862) for diarrhoea and a 95% credible interval of 0.670–1.107 (median: 0.860) for high fever. In addition, the incidence of diarrhoea in the intervention group was lower than that of the control group, with a probability of 86.7%, and the incidence of malaria was lower than that of the control group, with a probability of 88.6%. The total cost over the 10 years of period was estimated to $1,714,676, including start-up costs of $21,433 (1.3%), capital costs of $251,796 (14.7%) and recurrent costs of $1,441,448 (84%).

**Figure 6.**
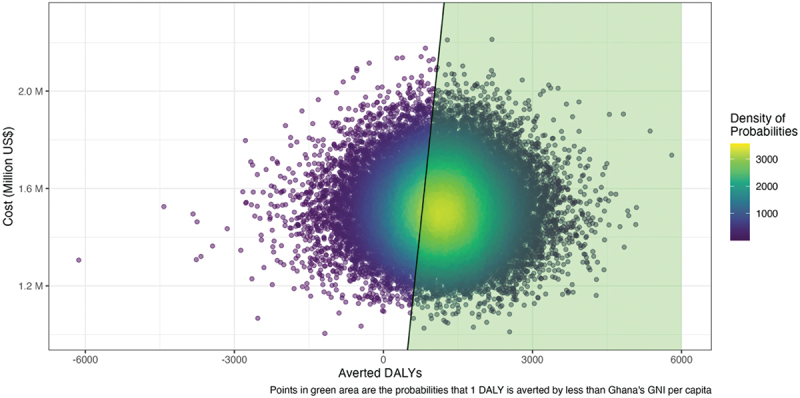
Cost-effectiveness plane.

Based on the statistical and economic models above, a cost-effectiveness analysis was performed by constructing 32,000 probability samples. In Figure 6, the cost-effectiveness analysis found that the 95% credible interval for the ICER ranged from -$720 to $2,919 (median: $1,150). The average ICER was $1,768, lower than Ghana’s 2016 GDP per capita of $1,931. We found a 65.3% probability of reducing 1 DALY at a cost less than GDP per capita. In Figure 6, the area marked in green is the section corresponding to probability samples that reduced 1 DALY at a lower cost than the GDP per capita.

**Figure 7.**
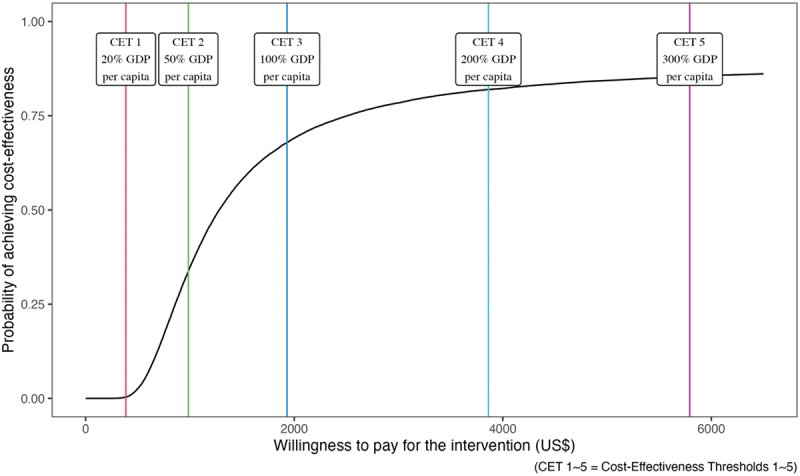
Cost-effectiveness acceptability curve.

We measured the probability of averting 1 DALY at a cost less than Ghana’s GDP per capita and accepting five different cost-effectiveness thresholds. In Figure 7, we plotted a cost-effectiveness acceptability curve and analysed which proportion of the 32,000 probability samples exceeded five different CETs. For the first CET, which was the most conservative (20% of GDP per capita), no sample exceeded the threshold. For the second CET (50% of GDP per capita), 26.5% of the probability samples crossed the threshold. For the third to fifth CETs (1 to 3 times GDP per capita), 65.3%, 81.4%, and 85.3% of the probability samples crossed the threshold. Starting with the third CET, the probability of exceeding the threshold was higher than the probability of not exceeding the threshold.

**Figure 8.**
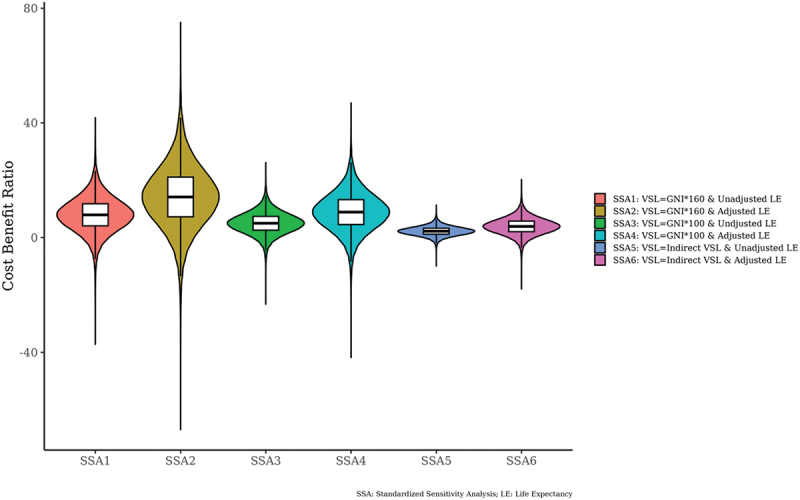
Violin plots on cost-benefit ratio.

**Figure 9.**
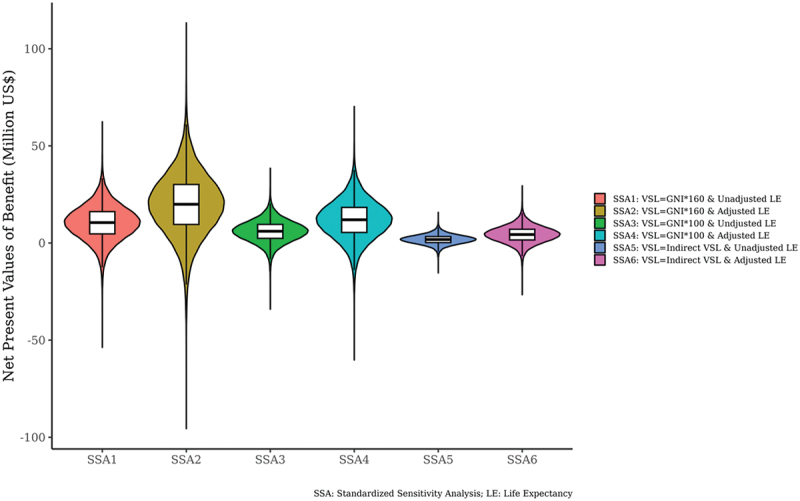
Violin plots on net present value of benefit.

Next, a cost-benefit analysis was conducted by converting health and non-health effects into benefits of monetary value. Overall, the mean and median of the cost-benefit ratio were 6.4 and 4.8, respectively. Of the 32,000 probability samples, 85.7% had a cost-benefit ratio of more than 1, and 65.8% had a cost-benefit ratio of more than 3. The median current value of the net benefit was $6.5 million. In Figures 8 and 9, a standardised sensitivity analysis (SSA) was conducted that changed the value of income elasticity, proportional life expectancy and the value of statistical life (VSL). In Figures 8 and 9, there were six different sensitivity analyses from SSA1 to SSA6, and even in the most pessimistic scenario (SSA 5), 76.6% of the 32,000 probability samples had a cost-benefit ratio of more than 1. The parameters in the sensitivity analysis can be found in the supplementary material #1.

## Discussion

In the cost-effectiveness analysis, specific CETs were applied to determine economic feasibility. The probability of satisfying the conservative first and second CETs was very low. However, there was a high possibility of satisfying the third to fifth CETs, which are commonly used. On the other hand, economic feasibility was consistently achieved in the cost-benefit analysis. In most probability samples, the cost-benefit ratio was greater than 1, and there was a high probability of the cost-benefit ratio exceeding 3. Even in the most pessimistic sensitivity analysis scenario, there was also a high probability that the cost-benefit ratio would be more than 1.

Previous research has shown that home visits for CHWs are affordable in low- and middle-income countries, saving the lives of newborns, lowering the incidence of HIV, and increasing exclusive breastfeeding [25–27]. The results of our study require careful interpretation. Activating CHVs in Ghana’s health system to induce disease reduction in children through home visits meets the WHO-CHOICE thresholds (third to fifth CET). However, those thresholds cannot be viewed as simple indicators of economic feasibility due to the lack of a rigorous analysis of Ghana’s national budget constraints [18]. Rather, the first and second CETs, which reflect common budget constraints in low- and middle-income countries, may have more realistic thresholds, and our study did not meet these thresholds. Therefore, we interpreted that the activation of CHVs only for childhood disease prevention could not necessarily achieve economic feasibility without additional budget support from donor countries. Similarly, the cost-benefit analysis shows that more benefits than costs are likely to occur, but the cost-benefit analysis should not be interpreted as indicative of absolute economic feasibility because it should be compared with other potential interventions that the policymaker in Ghana has. Also, the cost-benefit analysis only shows benefits without considering national budget constraints. Therefore, more research is needed to support the result of the cost-benefit analysis.

Nevertheless, this study has clear implications. First, CHVs’ home visits conducted solely to prevent childhood disease through behaviour change are not economically feasible within Ghana’s health system. Therefore, it seems that CHVs’ home visits should be combined with tasks whose economic feasibility has been verified. According to a previous field-based economic evaluation conducted in Ghana, CHVs’ intensive home visits after childbirth for newborn survival were economically feasible [25]. Therefore, to increase economic feasibility, CHVs’ home visits should be structured in a way that includes both tasks of childhood disease prevention and newborn survival.

Second, this study implies that in health systems like that of Ghana, where dual cadres of CHWs are operated, CHVs’ role in conducting effective home visits may have an indirect effect on reducing the burdens of CHOs. This demonstrates that CHVs are not a replaceable measure of CHOs; instead, they constitute a sustainable health workforce that works in harmony with CHOs. According to Ghana’s health policy, two CHOs should be deployed per CHPS zone, but in fact, only one CHO is working in 44% of CHPS zones [28]. Regional inequality is also serious. The staff availability ratio is 91% in Greater Accra, but only 53% in Volta [28]. A shortage of workforce causes a decrease in motivation and stress of health and medical workers in the field [29]. Currently, 7 out of 10 healthcare workers in Ghana, including CHOs, have moderate to severe stress from burnout [30]. Therefore, CHVs’ home visits play a significant role in responding to the problems caused by shortages of CHOs in Ghana.

These first and second implications could both assist in evidence-based policy-making that defines the optimal work scope of the dual cadres of CHWs in Ghana’s health system. This study also has implications for the global health community. To integrate CHW programmes with national health systems, we need to find the most effective scope of work for CHWs within national health systems. To this end, more economic evaluations of field experiment based CHW programmes need to be carried out. Ethiopia, for example, operates dual cadres of CHWs within its national health system (health extension workers and the Women’s Development Army), similar to the CHVs and CHOs of Ghana. Our study may provide ideas for future studies to specify the optimal scope of work for the dual cadres of CHWs in the Ethiopian setting.

This study has three main limitations. First, since this study used a probabilistic approach to cost-effectiveness and cost-benefit using Bayesian inference with weak prior information, the study’s results should not be interpreted as deterministic conclusions. Second, although this study used period prevalence as a proxy measure of incidence, there may be differences from the actual incidence in the population because the duration of diarrhoea and malaria onset is shorter than the longitudinal panel survey cycle. Third, using high fever as a proxy measure for malaria may overestimate the amount of DALYs, because high fever may be associated with other infections with low disability weights, such as respiratory diseases.

## Conclusion

Since conservative thresholds are more appropriate for low- and middle-income countries, we interpreted the results as indicating that activating CHV’s home visits to prevent childhood diseases did not satisfy economic feasibility. However, we found the potential economic strengths in the cost-benefit analysis, and the economic feasibility may be improved after integrating more tasks for CHV. To integrate CHW programmes with national health systems, we need to find the most effective scope of work for CHWs within national health systems.

## Supplementary Material

Supplemental Material

## Paper context

Activating community health volunteers in Ghana to induce disease reduction in children did not meet the realistic cost-effectiveness thresholds in low- and middle-income countries. Although, there was a high probability that one dollar’s investment in activating community health volunteers in Ghana would yield more than 3 dollars return in cost-benefit analysis. This study provides valuable insights to specify the optimal scope of work for the dual cadres of community health workers in the resource-scarce setting.

International Standard Randomized Controlled Trial Registry, ISRCTN49236178. https://doi.org/10.1186/ISRCTN49236178. Registered 16 May 2015 - Retrospectively registered.

## References

[1] Black RE, Taylor CE, Arole S, Bang A, Bhutta ZA, Chowdhury AMR, et al. Comprehensive review of the evidence regarding the effectiveness of community–based primary health care in improving maternal, neonatal and child health: 8. summary and recommendations of the expert panel. J Glob Health [Internet]. 2017;7:010908. 28685046 10.7189/jogh.07.010908 PMC5475312

[2] Jungo KT, Anker D, Wildisen L. Astana declaration: a new pathway for primary health care. Int J Public Health Internet. 2020;65:511–11. 32318780 10.1007/s00038-020-01368-5

[3] United Nations General Assembly. Resolution adopted by the general assembly on 10 October 2019: political declaration of the high-level meeting on universal health coverage. 2019.

[4] WHO. Global strategy on human resources for health: workforce 2030. 2016; 10.1186/s12960-024-00940-x PMC11450981 39363378

[5] Perry HB, Hodgins S. Health for the people: past, current, and future contributions of national community health worker programs to achieving global health goals. Global Health [Internet]. 2021;9:1–9. 10.9745/GHSP-D-20-00459 PMC8087430 33795359

[6] Haines A, de Barros EF, Berlin A, Heymann DL, Harris MJ. National UK programme of community health workers for COVID-19 response. Lancet (London, England) [Internet]. 2020;395:1173–1175. 32220277 10.1016/S0140-6736(20)30735-2 PMC7146683

[7] Hodgins S, Kok M, Musoke D, Lewin S, Crigler L, LeBan K, et al. Community health workers at the dawn of a new era: 1. Introduction: tensions confronting large-scale CHW programmes. Health Res Policy Syst [Internet]. 2021;19:109. 34641886 10.1186/s12961-021-00752-8 PMC8506102

[8] Nkonki L, Tugendhaft A, Hofman K. A systematic review of economic evaluations of CHW interventions aimed at improving child health outcomes. Hum Resour Health [Internet]. 2017;15:19. 28245839 10.1186/s12960-017-0192-5 PMC5331680

[9] Perry HB, Chowdhury M, Were M, LeBan K, Crigler L, Lewin S, et al. Community health workers at the dawn of a new era: 11. CHWs leading the way to “Health for All”. Health Res Policy Syst [Internet]. 2021;19. DOI:10.1186/s12961-021-00755-5. PMC8506098 34641891

[10] Nyonator FK, Awoonor-Williams JK, Phillips JF, Jones TC, Miller RA. The Ghana community-based health planning and services initiative for scaling up service delivery innovation. Health Policy Plann. 2005;20:25–34. 10.1093/heapol/czi003 15689427

[11] Ministry of Health. National community-based health planning and service (CHPS) policy. 2016

[12] Ghana’s community health officers and community health volunteers [Internet]. 2020. Available from: https://chwcentral.org/ghanas-community-health-officers-and-community-health-volunteers/.

[13] Diema Konlan K, Kossi Vivor N, Gegefe I, A Abdul-Rasheed I, Esinam Kornyo B, Peter Kwao I. The practice of home visiting by community health nurses as a primary healthcare intervention in a low-income rural setting: a descriptive cross-sectional study in the Adaklu district of the Volta Region, Ghana. The Sci World J [Internet]. 2021;2021:e8888845. 10.1155/2021/8888845 PMC8012147 33833622

[14] Chandi MG. Assessment of the implementation of the home visiting strategy: a case study of maternal and new born health care in the Ga south municipality of Ghana [Internet] [PhD thesis]. 2017. Available from: http://ugspace.ug.edu.gh/handle/123456789/23450.

[15] Bürkner P-C. Advanced Bayesian multilevel modeling with the R package brms. R J [Internet]. 2018 [cited 2023 Mar 19];10:395.

[16] Gabrio A, Baio G, Manca A. Bayesian statistical economic evaluation methods for health technology assessment. Oxford Research encyclopedia of economics and finance [Internet]. Oxford University Press; 2019 [cited 2023 Mar 19]. Available from: https://oxfordre.com/economics/view/10.1093/acrefore/9780190625979.001.0001/acrefore-9780190625979-e-451

[17] WHO. WHO guide for standardization of economic evaluations of immunization programmes. 2019.

[18] Woods B, Revill P, Sculpher M, Claxton K. Country-level cost-effectiveness thresholds: initial estimates and the need for further research. Value Health [Internet]. 2016;19:929–935. 27987642 10.1016/j.jval.2016.02.017 PMC5193154

[19] Robinson LA, Hammitt JK, Chang AY, Resch S. Understanding and improving the one and three times GDP per capita cost-effectiveness thresholds. Health Policy Plann [Internet]. 2017;32:141–145. 10.1093/heapol/czw096 27452949

[20] Bittl JA, He Y. Bayesian analysis: a practical approach to interpret clinical trials and create clinical practice guidelines. Circulation [Internet]. 2017;10. DOI:10.1161/CIRCOUTCOMES.117.003563 PMC6421843 28798016

[21] McNutt L-A, Wu C, Xue X, Hafner JP. Estimating the relative risk in cohort studies and clinical trials of common outcomes. Am J Epidemiol. 2003;157:940–943. 12746247 10.1093/aje/kwg074

[22] Population growth annual [Internet]. Available from: https://data.worldbank.org/indicator/SP.POP.GROW?locations=GH.

[23] Robinson LA, Hammitt JK, Cecchini M, Chalkidou K, Claxton K, Eozenou PH-V, et al. Reference case guidelines for benefit-cost analysis in global health and development. 126.

[24] Robinson LA, Hammitt JK, Jamison DT, Walker DG. Conducting benefit-cost analysis in low- and middle-income countries: introduction to the special issue. J Benefit-Cost Anal [Internet]. 2019;10:1–14. 33282627 10.1017/bca.2019.4 PMC7672367

[25] Pitt C, Tawiah T, Soremekun S, ten Asbroek AHA, Manu A, Tawiah-Agyemang C, et al. Cost and cost-effectiveness of newborn home visits: findings from the newhints cluster-randomised controlled trial in rural ghana. Lancet Glob Health [Internet]. 2016;4:e45–56. 26639857 10.1016/S2214-109X(15)00207-7 PMC5357735

[26] Thomas R, Probert WJM, Sauter R, Mwenge L, Singh S, Kanema S, et al. Cost and cost-effectiveness of a universal HIV testing and treatment intervention in zambia and south africa: evidence and projections from the HPTN 071 (PopART) trial. Lancet Glob Health [Internet]. 2021;9:e668–680. 33721566 10.1016/S2214-109X(21)00034-6 PMC8050197

[27] Tiruneh GT, Shiferaw CB, Worku A. Effectiveness and cost-effectiveness of home-based postpartum care on neonatal mortality and exclusive breastfeeding practice in low-and-middle-income countries: a systematic review and meta-analysis. BMC Pregnancy Childbirth. 2019;19:507. 31852432 10.1186/s12884-019-2651-6 PMC6921506

[28] Asamani JA, Ismaila H, Plange A, Ekey VF, Ahmed, A-M, Chebere M, et al. The cost of health workforce gaps and inequitable distribution in the ghana health service: an analysis towards evidence-based health workforce planning and management. Hum Resour Health [Internet]. 2021;19:43. 33789670 10.1186/s12960-021-00590-3 PMC8010987

[29] Dieleman M, Kane S, Zwanikken P, Gerretsen B, Organization WH. Realist review and synthesis of retention studies for health workers in rural and remote areas [Internet]. 2011. Available from: https://apps.who.int/iris/handle/10665/44548.

[30] Afulani P. COVID-19 has left Ghana’s healthcare workers stressed but simple things can help [Internet]. Available from: http://theconversation.com/covid-19-has-left-ghanas-healthcare-workers-stressed-but-simple-things-can-help-157907.

