# Supplementary material for: Trial-based economic evaluation of the system-integrated activation of community health volunteers in rural Ghana
Source: Glob Health Action. 2023 May 11;16(1):2203541. doi: 10.1080/16549716.2023.2203541 (PMC10177701; doi:10.1080/16549716.2023.2203541)
Supplement: Supplemental Material [file ZGHA_A_2203541_SM2450.zip › S1._Model_Parameters.docx]

Supplement Material #1. Model Parameters

Table 1. Parameters for Statistical Model (Beta distribution)

| **Name** | **Mean** | **Upper** | **Lower** | **Type** | **Alpha** | **Beta** | **Source** |
| --- | --- | --- | --- | --- | --- | --- | --- |
| Disability weight for diarrhea with mild severity | 0.074 | 0.104 | 0.049 | beta | 26 | 325 | GBD 2015 |
| Disability weight for diarrhea with moderate severity | 0.188 | 0.264 | 0.125 | beta | 23 | 99 | Ibid. |
| Disability weight for diarrhea with severe severity | 0.247 | 0.348 | 0.164 | beta | 21 | 63 | Ibid. |
| Disability weight for malaria with mild severity | 0.006 | 0.012 | 0.002 | beta | 5 | 846 | Ibid. |
| Disability weight for malaria with moderate severity | 0.051 | 0.074 | 0.032 | beta | 21 | 391 | Ibid. |
| Disability weight for malaria with severe severity | 0.133 | 0.190 | 0.088 | beta | 23 | 148 | Ibid. |

Table 2. Parameters for Statistical Model (Lognormal distribution)

| **Name** | **Mean** | **Upper** | **Lower** | **Type** | **Log(mean)** | **Log(sd)** | **Source** |
| --- | --- | --- | --- | --- | --- | --- | --- |
| Diarrhea Incidence | 2.055 | 2.443 | 1.675 | lognormal | 0.708 | 0.095 | GBD 2015 |
| Malaria Incidence | 0.433 | 0.582 | 0.264 | lognormal | -0.931 | 0.200 | Ibid. |

Table 3. Parameters for Statistical Model (Normal distribution)

| **Name** | **Mean** | **Upper** | **Lower** | **SD** | **Source** |
| --- | --- | --- | --- | --- | --- |
| Life expectancy at 2 years old | 65.9 | 67.2 | 64.5 | 0.688 | Max Roser et al. (2013) |

Table 4. Parameters for Cost Model (from the project expenditure record)

| **Type1** | **Type2** | **Account** | **Account2** | **Quantity** | **Life span** | **Unit Cost ($)** | **Unit Cost (ghs)** |
| --- | --- | --- | --- | --- | --- | --- | --- |
| initial cost | financial | CHV recruitment | community consultation | 1 |  | 6,445.01 | 25,200.00 |
| initial cost | financial | CHV recruitment | community durbar  (9 villages) | 9 |  | 179.03 | 700.00 |
| initial cost | financial | CHV recruitment | workshop and printing recruitment brochure | 1 |  | 2,046.04 | 8,000.00 |
| initial cost | financial | CHV initial training (10 days) | conference hall rent | 1 |  | 639.39 | 2,500.00 |
| initial cost | financial | CHV initial training (10 days) | meal | 90 |  | 38.36 | 150.00 |
| initial cost | financial | CHV initial training (10 days) | lecturer daily allowance | 10 |  | 255.75 | 1,000.00 |
| initial cost | financial | CHV initial training (10 days) | lecturer transportation | 10 |  | 51.15 | 200.00 |
| initial cost | financial | CHV initial training (10 days) | snack | 90 |  | 10.23 | 40.00 |
| initial cost | financial | CHV initial training (10 days) | water | 90 |  | 3.84 | 15.00 |
| initial cost | financial | CHV initial training (10 days) | transportation allowance | 82 |  | 25.58 | 100.00 |
| initial cost | financial | CHV initial training (10 days) | media coverage | 1 |  | 805.63 | 3,150.00 |
| capital cost | financial | equipment | field monitoring vehicle | 1 | 5 | 38,363.17 | 150,000.00 |
| capital cost | financial | equipment | furniture, electronics, and computer | 1 | 5 | 1,636.83 | 6,400.00 |
| capital cost | financial | CHV activity kits | raincoat | 82 | 2 | 14.07 | 55.00 |
| capital cost | financial | CHV activity kits | flashlight | 82 | 2 | 5.12 | 20.00 |
| capital cost | financial | CHV activity kits | backpack | 82 | 2 | 14.07 | 55.00 |
| capital cost | financial | CHV activity kits | t-shirts | 82 | 2 | 4.68 | 18.29 |
| capital cost | financial | CHV activity kits | id card | 82 | 2 | 2.56 | 10.00 |
| capital cost | financial | CHV activity kits | certificate | 82 | 2 | 0.77 | 3.00 |
| capital cost | financial | CHV activity kits | plastic folder | 82 | 2 | 0.77 | 3.00 |
| capital cost | financial | CHV activity kits | logbook | 82 | 2 | 12.79 | 50.00 |
| capital cost | financial | CHV activity kits | activity guidebook | 82 | 2 | 28.07 | 109.76 |
| capital cost | financial | CHV activity kits | digital thermometer | 82 | 2 | 7.67 | 30.00 |
| capital cost | financial | CHV activity kits | visual educational materials | 82 | 2 | 42.11 | 164.63 |
| recurrent cost | financial | CHV activity consumables | flashlight batteries | 82 |  | 7.67 | 30.00 |
| recurrent cost | financial | CHV activity consumables | notepad | 82 |  | 2.05 | 8.00 |
| recurrent cost | financial | CHV activity consumables | pen/pencil/eraser/sharpener | 82 |  | 0.77 | 3.00 |
| recurrent cost | financial | medical supplies | ORS | 82 |  | 30.69 | 120.00 |
| recurrent cost | financial | medical supplies | aqua tablet | 82 |  | 30.69 | 120.00 |
| recurrent cost | financial | medical supplies | malaria rdt | 82 |  | 122.76 | 480.00 |
| recurrent cost | financial | communication | CHV’s mobile phone credit | 82 |  | 15.35 | 60.00 |
| recurrent cost | financial | communication | CHN/O’s mobile phone credit | 20 |  | 30.69 | 120.00 |
| recurrent cost | financial | motivation | CHV transportation support | 82 |  | 30.69 | 120.00 |
| recurrent cost | financial | motivation | CHV foods | 82 |  | 92.07 | 360.00 |
| recurrent cost | financial | motivation | best CHV award | 13 |  | 59.02 | 230.77 |
| recurrent cost | financial | motivation | best community award | 3 |  | 1,364.02 | 5,333.33 |
| recurrent cost | financial | supportive supervision | monthly review meeting | 12 |  | 204.60 | 800.00 |
| recurrent cost | financial | supportive supervision | bi-annual workshop | 2 |  | 1,278.77 | 5,000.00 |
| recurrent cost | financial | supportive supervision | outreach monitoring | 12 |  | 724.64 | 2,833.33 |
| recurrent cost | financial | supportive supervision | fuel and car maintenance | 12 |  | 427.73 | 1,672.42 |
| recurrent cost | financial | administration | project office utilities | 12 |  | 980.39 | 3,833.33 |
| recurrent cost | financial | administration | office rent | 12 |  | 511.51 | 2,000.00 |
| recurrent cost | financial | personnel | staffs (international) | 2 |  | 40,920.72 | 160,000.00 |
| recurrent cost | financial | personnel | staffs (local) | 1 |  | 5,728.90 | 22,400.00 |
| recurrent cost | financial | personnel | driver | 1 |  | 4,092.07 | 16,000.00 |
| recurrent cost | economic | personnel | CHV's opportunity cost for time (20% of working time) | 82 |  | 95.14 | 372.00 |
| recurrent cost | economic | personnel | caregiver's opportunity cost for time (30 mins every quarter) | 2,476 |  | 0.33 | 1.27 |

* All financial cost parameters were derived from the project expenditure records, and all economic costs were derived by multiplying time-spent for home visitation and informal hourly wage of Ghana

Table 5. Parameters for Economic Evaluation Model

| **Name** | **Value** | **Name** | **Value** | **Name** | **Value** | **Name** | **Value** | **Source** |
| --- | --- | --- | --- | --- | --- | --- | --- | --- |
| Transition probability of diarrhea (mild) | 0.624 | Transition probability of diarrhea (moderate) | 0.289 | Transition probability of diarrhea (severe) | 0.042 | Case Fatality Rate of diarrhea | 0.0005 | GBD 2015 |
| Transition probability of malaria (mild) | 0.500 | Transition probability of malaria (moderate) | 0.300 | Transition probability of malaria (severe) | 0.200 | Case Fatality Rate of malaria | 0.024 | GBD 2015 & Author’s assumption |
| Duration of diarrhea  (days) | 4.2 | Duration of malaria  (days) | 7 | CHV’s annual attrition rate | 0.083 | Discount Rate | 0.03 | Abbey et al. 2014 & Author’s assumption |

Table 6. Standardized Sensitivity Analysis for Cost-Benefit Analysis

|  | Income elasticity = 1.0 | Proportional LE (Life Expectancy)  (LE at birth / LE at 35 age) |
| --- | --- | --- |
| VSL extrapolated from an OECD VSL-to-GNI per capita ratio of 160 | SSA1 | SSA2 |
| VSL extrapolated from a U.S. VSL-to-GNI per capita ratio of 100 | SSA3 | SSA4 |
| VSL extrapolated from a U.S. VSL of $9.4 million and U.S. GNI per capita of $57,900 | SSA5 | SSA6 |
